# A multicenter evaluation of computable phenotyping approaches for SARS-CoV-2 infection and COVID-19 hospitalizations

**DOI:** 10.1038/s41746-022-00570-4

**Published:** 2022-03-08

**Authors:** Rohan Khera, Bobak J. Mortazavi, Veer Sangha, Frederick Warner, H. Patrick Young, Joseph S. Ross, Nilay D. Shah, Elitza S. Theel, William G. Jenkinson, Camille Knepper, Karen Wang, David Peaper, Richard A. Martinello, Cynthia A. Brandt, Zhenqiu Lin, Albert I. Ko, Harlan M. Krumholz, Benjamin D. Pollock, Wade L. Schulz

**Affiliations:** 1 Section of Cardiovascular Medicine, Department of Internal Medicine, Yale School of Medicine, New Haven, CT USA; 2 Center for Outcomes Research and Evaluation, Yale-New Haven Hospital, New Haven, CT USA; 3 Computer Science & Engineering, Texas A&M University, College Station, TX USA; 4 Section of General Internal Medicine, Yale School of Medicine, New Haven, CT USA; 5 Department of Health Policy and Management, Yale School of Public Health, New Haven, CT USA; 6 Division of Health Care Delivery Research, Robert D. and Patricia E. Kern Center for the Science of Health Care Delivery, Mayo Clinic, Rochester, MN USA; 7 Division of Clinical Microbiology, Mayo Clinic Rochester, Rochester, MN USA; 8 Department of Quality, Experience, and Affordability, Mayo Clinic, Rochester, MN USA; 9 Equity Research and Innovation Center, General Internal Medicine, Yale School of Medicine, New Haven, CT USA; 10 Center for Medical Informatics, Yale School of Medicine, New Haven, CT USA; 11 Department of Laboratory Medicine, Yale School of Medicine, New Haven, CT USA; 12 Section of Infectious Diseases, Department of Internal Medicine, Yale School of Medicine, New Haven, CT USA; 13 VA Connecticut Healthcare System, West Haven, CT USA; 14 Department of Epidemiology of Microbial Diseases, Yale School of Public Health, New Haven, CT USA; 15 Instituto Gonçalo Moniz, Fundação Oswaldo Cruz, Salvador, BA Brazil

**Keywords:** Outcomes research, Public health, Viral infection, Epidemiology

## Abstract

Diagnosis codes are used to study SARS-CoV2 infections and COVID-19 hospitalizations in administrative and electronic health record (EHR) data. Using EHR data (April 2020–March 2021) at the Yale-New Haven Health System and the three hospital systems of the Mayo Clinic, computable phenotype definitions based on ICD-10 diagnosis of COVID-19 (U07.1) were evaluated against positive SARS-CoV-2 PCR or antigen tests. We included 69,423 patients at Yale and 75,748 at Mayo Clinic with either a diagnosis code or a positive SARS-CoV-2 test. The precision and recall of a COVID-19 diagnosis for a positive test were 68.8% and 83.3%, respectively, at Yale, with higher precision (95%) and lower recall (63.5%) at Mayo Clinic, varying between 59.2% in Rochester to 97.3% in Arizona. For hospitalizations with a principal COVID-19 diagnosis, 94.8% at Yale and 80.5% at Mayo Clinic had an associated positive laboratory test, with secondary diagnosis of COVID-19 identifying additional patients. These patients had a twofold higher inhospital mortality than based on principal diagnosis. Standardization of coding practices is needed before the use of diagnosis codes in clinical research and epidemiological surveillance of COVID-19.

## Introduction

The COVID-19 pandemic has led to the rapid adoption of real-world evidence to guide the treatment of and the public health response to a novel pathogen^1–5^. The identification of both SARS-CoV-2 infection and COVID-19 hospitalization is of current clinical and regulatory importance given the need for case identification for epidemiologic surveillance to track the infections, mortality, and vaccine effectiveness. Similarly, clinical predictive models that rely on appropriate case classification and studies that track the long-term effects of SARS-CoV-2 infection may be biased if case definitions are inaccurate or capture only subsets of individuals infected with SARS-CoV-2. Administrative data represent a widely available real-world data (RWD) source to monitor COVID-19 cases and hospitalizations using diagnosis codes.

Administrative data, a source of RWD generated from billing claims, can be used for disease surveillance, to follow hospitalization rates, and characterize patient outcomes on a large scale as well as evaluate the effects of health policy for these measures^6–10^. However, claims could also represent individuals tested for infection rather those with actual disease, thereby biasing the study of epidemiological investigations, as has been shown in other conditions^11,12^. To ensure that high-quality data guide national policy and biomedical research, there is a need to evaluate the accuracy of the diagnostic code-based approaches used to define cases of SARS-CoV-2 infection and hospitalization.

The adoption of health information technology systems has positioned health systems to improve case identification by incorporating more detailed clinical data from the electronic health record (EHR) with diagnosis codes, which allows for the development of more accurate computable phenotypes, as well as the validation of computable phenotyping approaches based on diagnosis codes^13–19^.

In this study from two large health systems with academic and community-based practices, we evaluate the accuracy of various approaches to identify people with SARS-CoV-2 infection and COVID-19 hospitalizations based on diagnostic codes and laboratory testing results from the EHR. We also assess how cohort definitions affect the evaluation of outcomes through an assessment of inhospital mortality across these cohorts.

## Results

### SARS-CoV-2 testing and diagnosis rates

There were 69,423 individuals with either a diagnosis of COVID-19 or a positive PCR for SARS-CoV-2 infection in the Yale-New Haven Health System between April 1, 2020 and March 1, 2021. During this period, there were 75,748 SARS-CoV2 infections identified across the three Mayo Clinic sites. At Yale, the mean age of patients was 46.0 (±22.4) years and 45.0% of patients were men. Nearly one fourth of patients were of Hispanic ethnicity (22.8%), 57.6% of patients had a recorded race of White and 15.2% were Black (Table 1). In contrast, patients in the Mayo Clinic were younger (mean age, 41.8 ± 20.6 years) and were predominantly White (80.6% vs. 4.0% Black) (Table 1).

**Table 1:** Characteristics of patients across mutually exclusive computable phenotypes from the Yale New-Haven Health System and Mayo Clinic.

Characteristics	Overall	Diagnosis PLUS PCR/Antigen+	Diagnosis only	PCR/Antigen+ only
*Yale New Haven Health System*				
Number of patients	69423	41,955	19,068	8400
Age (mean (SD))	46.0 (22.4)	51.2 (23.8)	52.4 (24.6)	42.6 (20.7)
Men, *n* (%)	31271 (45.0)	19,300 (46)	8335 (43.7)	3636 (43.3)
Race, *n* (%)				
Black	10,582 (15.2)	7219 (17.2)	732 (12.1)	1171 (13.9)
White	39,976 (57.6)	22,462 (53.5)	4221 (70.0)	4320 (51.4)
Asian	1248 (1.8)	732 (1.7)	87 (1.4)	144 (1.7)
Native Hawaiian/Other Pacific Islander	242 (0.3)	151 (0.4)	11 (0.2)	37 (0.4)
American Indian or Alaska Native	144 (0.2)	83 (0.2)	10 (0.2)	20 (0.2)
Other race	11,833 (17.0)	8207 (19.6)	619 (10.3)	1586 (18.9)
Unknown	5398 (7.8)	3101 (7.4)	354 (5.9)	1122 (13.4)
Hispanic ethnicity (%)	15,829 (22.8)	11,037 (26.3)	838 (13.9)	2072 (24.7)
*Mayo Clinic (all three sites)*				
Number of patients	75,748	46,522	2455	26,771
Age (mean (SD))	41.8 (20.6)	54.8 (22.2)	58.2 (22.3)	33.5 (16.4)
Men, *n* (%)	37,340 (49.3)	22,475 (48.3)	1225 (49.9)	13,640 (51.0)
Race, *n* (%)				
Black	3064 (4.0)	2395 (5.1)	110 (4.5)	569 (2.1)
White	61,063 (80.6)	37,161 (79.9)	2070 (84.3)	21,832 (81.6)
Asian	1685 (2.2)	1218 (2.6)	50 (2)	411 (1.5)
Native Hawaiian/Other Pacific Islander	115 (0.2)	85 (0.2)	0 (0)	22 (0.1)
American Indian or Alaska Native	353 (0.5)	249 (0.5)	45 (1.8)	53 (0.2)
Other race	3177 (4.2)	2403 (5.2)	78 (3.2)	696 (2.6)
Unknown	6291 (8.3)	3010 (6.5)	93 (3.8)	3188 (11.9)
Hispanic ethnicity (%)	6057 (8.0)	4603 (9.9)	216 (8.8)	1238 (4.6)

### Computable phenotype accuracy for SARS-CoV-2 infection at Yale

**Fig. 1:**
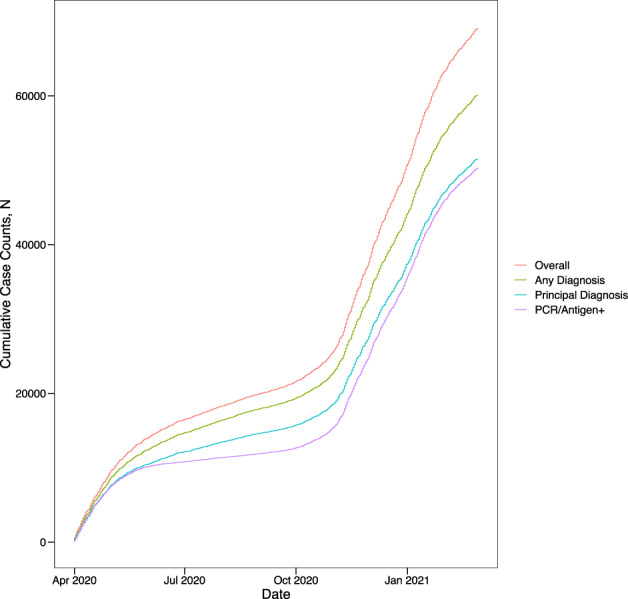
SARS-CoV2 case counts by phenotyping strategy. The absolute cumulative SARS-CoV-2 cases by adjudication strategy across the study period. The cases are based on either principal diagnosis or any diagnosis, compared with a polymerase chain reaction or antigen test for SARS-CoV-2.

Of the 69,423 individuals included in our Yale cohort, 61,023 (87.9%) had a diagnosis of COVID-19 in the EHR and 50,355 (72.7%) had a positive SARS-CoV-2 PCR or antigen test. There were consistent differences in number of SARS-CoV-2 infections based on the diagnosis and laboratory-based phenotyping strategies throughout the study period, with diagnostic codes being more common than positive laboratory test findings (Fig. 1). Similar patterns were observed for non-specific coronavirus diagnoses (Supplementary Table 1 in the Online Supplementary).

**Fig. 2:**
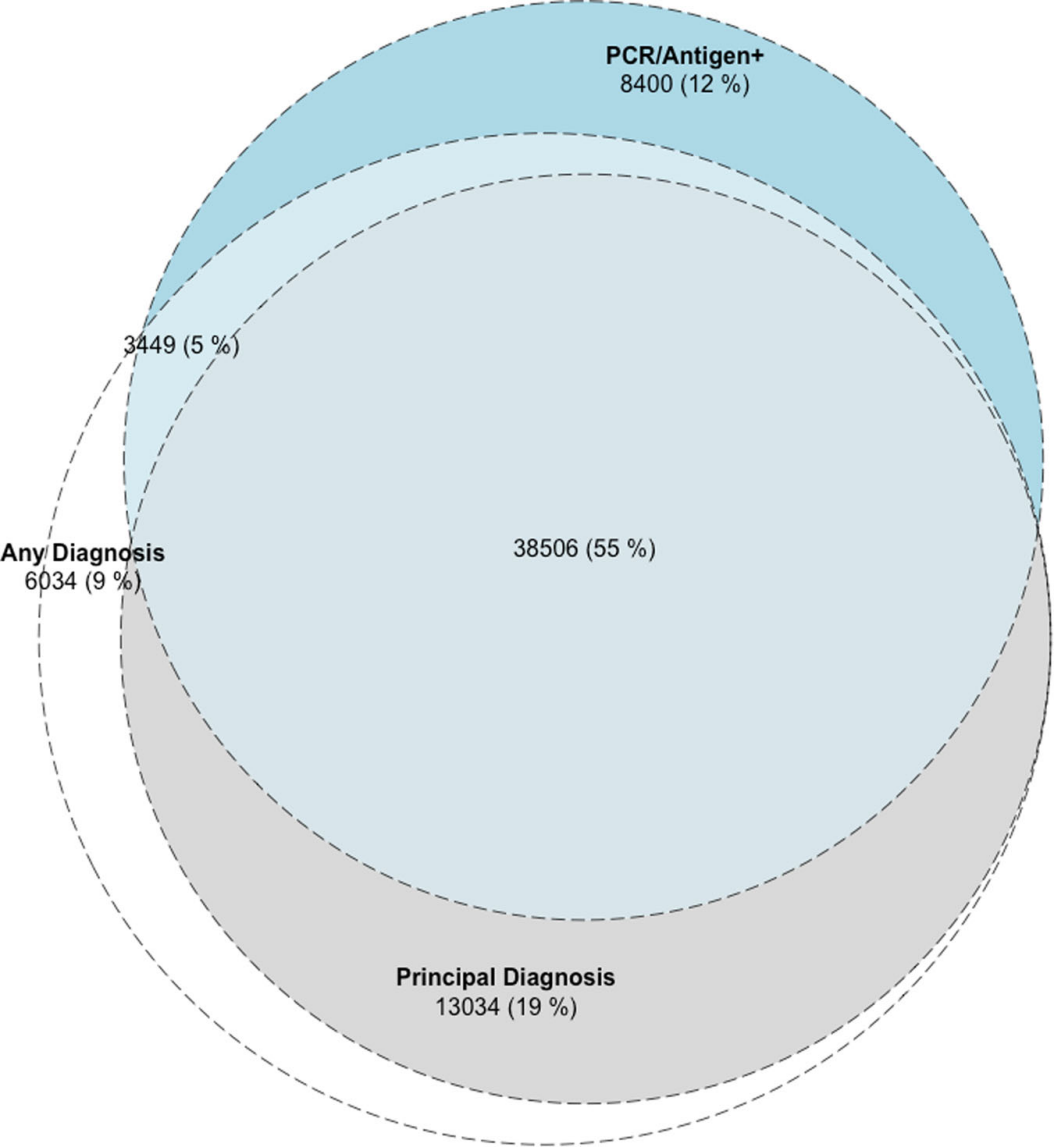
Overlap of SARS-CoV2 case counts by computational phenotyping strategies. Computable phenotypes for SARS-CoV-2 infection across the study period at Yale New Haven Health System.

Of the 50,355 patients with a positive laboratory test for SARS-CoV-2, 41,995 (83.3%) had a diagnosis of COVID-19 recorded in the EHR (Fig. 2). Moreover, there were 19,068 patients (31.2%) who had a COVID-19 diagnosis without a positive lab test for SARS-CoV-2. The characteristics of patients in these groups are included in Table 1.

In a manual chart review of a random sample of 30 patients with a diagnosis and without a positive SARS-CoV-2 test, all had a healthcare visit for SARS-CoV-2 testing but with a subsequent negative laboratory test.

The use of a diagnosis code of COVID-19 as the criteria to identify SARS-CoV-2 infection had a precision (or positive predictive value) of 68.8% (95% CI, 68.4% to 69.1%) and recall (or sensitivity) of 83.3% (95% CI, 83.0% to 83.6%).

**Fig. 3:**
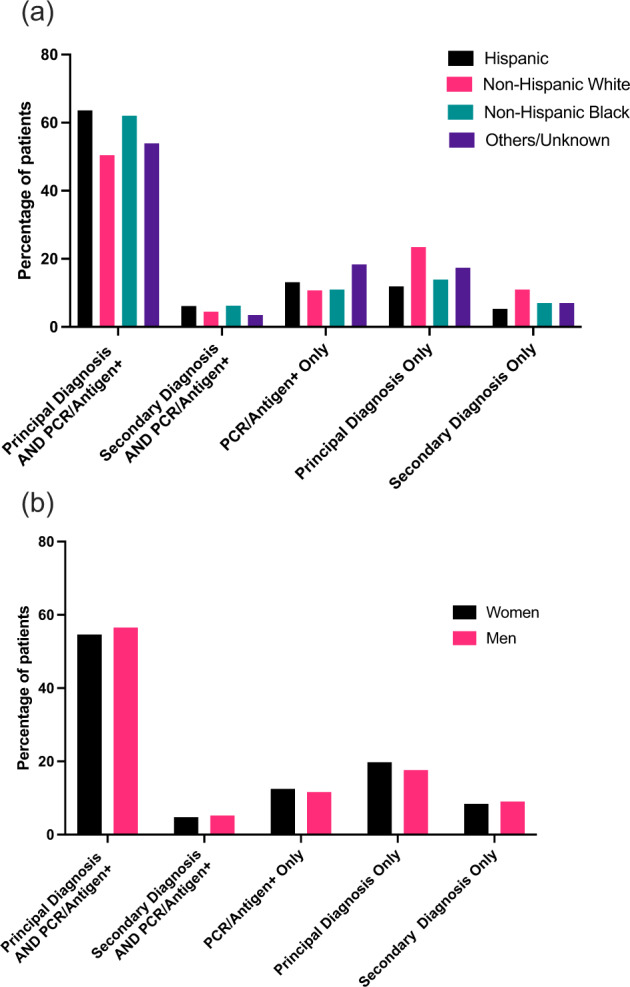
Demographic differences in SARS-CoV2 phenotyping strategies. Computable phenotypes for SARS-CoV-2 infection by **a** Race/Ethnicity and **b** Sex in the Yale-New Haven Health System.

There were significant differences in concordance between patient identification strategies during the study period (Supplementary Fig. 1). Among patients with either a diagnosis code for COVID-19 or a laboratory diagnosis of SARS-CoV-2, 51% of patients had both a diagnosis code and a positive laboratory test between April and August 2020, while 65% of patients had both present between September 2020 and March 2021 (*P* < 0.001). There were modest differences across racial and ethnic groups (Fig. 3a). Among Hispanics and non-Hispanic Black patients, 69.5% and 68.7% patients, respectively had both a diagnosis of COVID-19 and a positive laboratory test, compared with 54.5% of non-Hispanic White patients. There was also a significant difference by sex, with more women having a concomitant diagnosis code and positive laboratory test than men (61.4% vs. 59.1%, *P* < 0.001 for all) (Fig. 3b).

### Accuracy of phenotypes for SARS-CoV-2 infections across Mayo Clinic sites

**Fig. 4:**
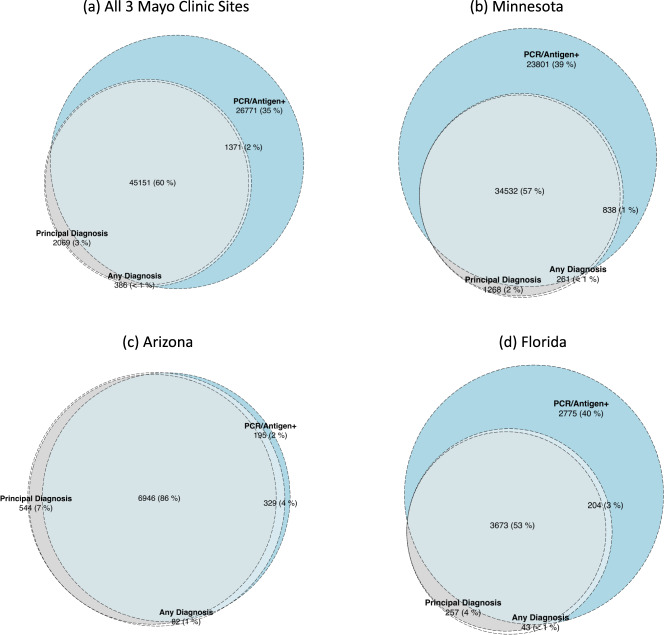
SARS-CoV2 case counts by computational phenotyping strategies in the Mayo Clinic System. Computable phenotypes for SARS-CoV-2 infection across the study period at the Mayo Clinic System, **a** across all Mayo Clinic sites, **b** Rochester, **c** Arizona, and **d** Florida.

At Mayo Clinic, a diagnosis of COVID-19 was associated with high precision for SARS-CoV2 infection (95.3%, Fig. 4). However, the recall (or sensitivity) was low (63.3%). Further, there was substantial variation across the Mayo Clinic sites, with the sensitivity of a COVID-19 diagnosis identifying SARS-CoV-2 infection varying between 59.2% in Rochester to 97.3% in Arizona (Fig. 4).

### Computable phenotype accuracy for COVID-19 hospitalization at Yale

Based on visit start date, there were a total of 5555 discharges using our overall phenotyping strategy from April 1, 2020 and January 31, 2021 at Yale. Of these, 5109 (92.0%) discharges had a principal diagnosis of COVID-19 and the remaining 446 had a principal diagnosis for a COVID-19 related severe presentation and a secondary diagnosis of COVID-19 on the same visit. Finally, there were 343 individuals who had a secondary, but not primary, diagnosis of COVID-19 which were excluded from analysis as these diagnoses were incidental findings or hospital-acquired infections. Those with a COVID-19 primary diagnosis were less frequently male (50.9% vs. 59.6%, *P* < 0.001) and were more frequently Black (20.9% vs. 17.3%, *P* = 0.02).

The vast majority of patients had a positive SARS-CoV-2 PCR or antigen test during their hospitalization across both patients hospitalized with a COVID-19 primary diagnosis (94.8%, *n* = 4843) or a secondary diagnosis (91.9%, *n* = 410). A manually abstracted sample of ten charts of hospitalized individuals without a positive laboratory test but with a principal diagnosis of COVID-19 found that seven of these patients had a positive COVID-19 test at another healthcare facility prior to presentation and three had a strong clinical suspicion for COVID-19 but a negative PCR test.

### COVID-19 hospitalization phenotypes across Mayo Clinic sites

A smaller proportion of patients with a principal diagnosis of COVID-19 in the Mayo Clinic System, as compared with those at Yale, had a positive SARS-CoV-2 PCR or antigen test during the hospitalization (80.5%, *n* = 2378), but the proportion was similar between Mayo Clinic and Yale for patients with a secondary diagnosis (90.7%, 331). A manually abstracted sample of ten charts among individuals who had a principal diagnosis of COVID-19 without a positive laboratory test identified that nine of these patients had a positive SARS-CoV-2 test at another healthcare facility prior to presentation and one did not have a documented SARS-CoV-2 test.

### Relationship between COVID-19 hospitalization phenotype definition and inhospital mortality rate

**Fig. 5:**
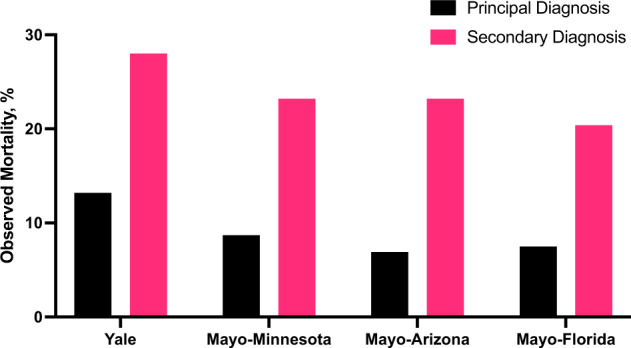
Mortality for COVID-19 hospitalizations defined by principal and secondary diagnosis by study site. Mortality represents inhospital death and discharge to hospice from index admission.

At Yale, the inhospital mortality rate for those hospitalized with a principal diagnosis of COVID-19 was 13.2% (675 of 5109), which was significantly lower than those with a secondary diagnosis of COVID-19 and related primary diagnosis code for sepsis or respiratory failure, who had an inhospital mortality that was nearly double (28.0%, 125 of 446, *P* < 0.001) (Table 2). This pattern was also observed at Mayo Clinic, with an 8.0% (237 of 2954) inhospital mortality rate among patients with a principal diagnosis of COVID-19, compared with 22.7% (83 of 365) among those with a secondary diagnosis of COVID-19. This was observed across all three Mayo Clinic sites (Fig. 5).

**Table 2:** Characteristics of hospitalized COVID-19 patients with a principal or secondary diagnosis of COVID-19 (U07.1).

Characteristics	Overall	Principal diagnosis of COVID-19	Secondary diagnosis of COVID-19^a^
*Yale New Haven Health System*			
Number of patients	5555	5109	446
Age (mean (SD))	66.37 (17.59)	66.17 (17.68)	68.63 (16.44)
Men, *n* (%)	2867 (51.6)	2601 (50.9)	266 (59.6)
Race, *n* (%)			
Black	1145 (20.6)	1068 (20.9)	77 (17.3)
White	3156 (56.8)	2880 (56.4)	276 (61.9)
Asian	103 (1.9)	96 (1.9)	<10 (1.6)
Native Hawaiian/other			
Pacific Islander	19 (0.3)	19 (0.4)	0 (0.0)
American Indian or Alaska			
Native	12 (0.2)	11 (0.2)	<10 (0.2)
Other race	1043 (18.8)	960 (18.8)	83 (18.6)
Unknown	77 (1.4)	75 (1.5)	<10 (0.4)
Hispanic ethnicity (%)	1243 (22.4)	1152 (22.5)	91 (20.4)
Inhospital mortality/discharge to Hospice, *n* (%)	800 (14.4)	675 (13.2)	125 (28.0)
*Mayo Clinic*			
Number of patients	3319	2954	365
Age (mean (SD))	65.47 (17.84)	65.41 (17.98)	65.92 (16.64)
Men, *n* (%)	1893 (57.0)	1659 (56.6)	234 (64.1)
Race, *n* (%)			
Black	173 (5.2)	149 (5.0)	24 (6.6)
White	2714 (81.8)	2427 (82.2)	287 (76.6)
Asian	110 (3.3)	93 (3.2)	17 (4.7)
Native Hawaiian/other			
Pacific Islander	<10 (0.2)	<10 (0.2)	0 (0.0)
American Indian or Alaska			
Native	116 (3.5)	100 (3.4)	16 (4.4)
Other race	126 (3.8)	109 (3.7)	‘17 (4.7)
Unknown	74 (2.2)	70 (2.4)	4 (1.1)
Hispanic ethnicity, *n* (%)	326 (9.8)	277 (9.4)	49 (13.4)
Inhospital mortality/discharge to Hospice, *n* (%)	320 (9.6)	237 (8.0)	83 (22.7)

^a^With a principal diagnosis for respiratory failure, sepsis, or pneumonia.

## Discussion

In two large, integrated health systems with multiple care delivery networks and associated outpatient clinics, COVID-19 diagnosis codes alone were frequently not consistent with case identification and epidemiological surveillance of SARS-CoV-2 infection based on antigen/PCR testing, with significant variation across two major health systems. In contrast, nearly all patients with a principal diagnosis of COVID-19 were admitted with a SARS-CoV2 infection, though a focus on principal diagnosis alone would miss an additional 10% of patients with a clinical profile consistent with severe COVID-19, which were recorded as a secondary diagnosis of COVID-19. The latter group had an over twofold higher mortality rate compared with patients with a principal diagnosis of COVID-19.

This study extends the literature in several key ways. The study leverages multiple EHRs as a source of RWD, rather than administrative claims, and evaluates the accuracy of diagnostic codes for SARS-CoV-2 case identification across outpatient and inpatient healthcare settings. Moreover, in addition, we evaluated the association of phenotype definitions on inferred short-term outcomes. A previous study found that the COVID-19 diagnosis code, U07.1 was rapidly incorporated into the workflow of US hospitals in early 2020^20^, and among hospitalized patients, had a high sensitivity and specificity of the code for laboratory confirmed disease. However, that study was limited in using data only through May 2020, with only 4965 SARS-CoV-2 positive laboratory tests. Moreover, the accuracy measures were driven by the 89.6% of the cohort that did not have either a positive test or a diagnosis code for COVID-19^20^. The evaluation of COVID-19 diagnosis codes also focused exclusively among hospitalized patients and did not evaluate the role of diagnosis codes in case surveillance.

We confirm that an inpatient diagnosis of COVID-19 has retained a large positive predictive value for clinical COVID-19. Yet, we found significant heterogeneity in outcomes based on whether COVID-19 was included as a principal or a secondary diagnosis. Finally, we evaluated the approach across two different hospital systems spanning four distinct geographic regions over an entire year. Through a limited exploratory expert chart review, we obtained some additional insights about discordant diagnosis codes and laboratory results. Specifically, we found that in the reviewed cases of outpatient COVID-19 diagnosis codes, the codes corresponded to diagnoses captured for subsequently negative laboratory tests, rather than missed laboratory tests at external sites. Moreover, in the inpatient setting, COVID-19 diagnosis codes without a laboratory test were associated with either a positive test at another institution or a high clinical suspicion for the disease without a positive test.

There are many possible reasons for the incorrect classification of SARS-CoV-2 infections by diagnosis code. Many studies have shown the apparent inaccuracy of various EHR data elements, such as the clinical history and problem list^21,22^. Clinical uncertainty related to a diagnosis, potential stigma associated with the addition of a diagnosis to the medical record, clinical workflows that do not promote the capture of structured data elements, and miscoded diagnoses can all impact the ability to define a digital phenotype that accurately identifies patients^21,23,24^. Moreover, diagnosis codes are often included when evaluating a suspected condition and may be misconstrued as proof of diagnosis, particularly in data captured in near real-time.

Our study finds evidence of the changing sensitivity of the codes over the study period, with fewer diagnoses associated with clinical tests in the early phase of the pandemic. This is consistent with the limited availability of diagnostic tests in that period. Moreover, our racially and ethnically diverse study population allowed our study to specifically evaluate differential performance of diagnosis codes for case surveillance in racial/ethnic minority groups and women, especially due to the disproportionately large effect of the pandemic on racial/ethnic minorities^25^. We found modest differences in performance of codes across these groups, with a lower performance of diagnosis codes in non-Hispanic White patients relative to Hispanic and non-Hispanic Black individuals. While the mechanism for these differences is unclear, it could represent a combination of the larger burden of SARS-CoV2 infection and COVID-19 hospitalizations among Black and Hispanic individuals, and the broader availability of testing through community-based practices to individuals from the White communities, with more visits for clinical suspicion for COVID-19.

Our study also highlights the value of health information systems in disease monitoring through logical cohort definitions, which can be explicitly confirmed across different data elements. This was manifested in our ability to assess the reliability of diagnosis codes through a large period in the pandemic when, contrary to expectations, increased access to laboratory testing during the later phase of the pandemic did not eliminate the difference between the relative prevalence of diagnosis codes and laboratory testing, suggesting the role of coding practices rather than access to testing. Moreover, our work supports the need for continuous monitoring and validation of computable phenotypes, especially those that rely solely on diagnosis codes, such as those used to analyze administrative data sets. The variation in the reliability of diagnosis codes may represent a combination of differences in care practices, including the clinical threshold for testing for SARS-CoV-2 due to either accessibility of testing or clinical protocols, and the threshold for using diagnosis codes for documenting care for patients with a clinical suspicion.

Our study has several limitations. First, while our study evaluates the accuracy of diagnosis codes in the study of the clinical epidemiology of SARS-CoV-2 infection and COVID-19 hospitalizations based on a concomitant positive test, the true population of interest is likely larger^16,26^, and could include with clinical manifestation of disease without either a positive laboratory test or a diagnosis code. While of interest, these populations are challenging to define using existing computational phenotyping strategies.

Second, while we focused on two broad interconnected health systems and affiliated laboratories and receive testing information from laboratories that exchange data via the Epic EHR, not all external laboratory data were available from testing in the outpatient setting. However, in manual chart review of a sample of patients with an outpatient diagnosis of COVID-19 without a reported positive PCR or antigen test, all such records were for patients undergoing SARS-CoV-2 testing with the diagnosis assigned for the clinical or laboratory encounter to obtain the test. Third, while both study sites used data from the Epic EHR, variation in care practices, coding conventions, and EHR engagement strategies would likely introduce variation in how diagnosis codes and laboratory testing are recorded, To ensure that the data elements for current study corresponded to the same clinical entities, the data extraction was harmonized through direction from local expert clinicians and informaticians with experience with working with their respective data sources.

Fourth, we cannot infer coding practices at other institutions not included in the study. However, the two large integrated multi-hospital health systems included in the study demonstrated substantial inter-hospital heterogeneity in coding practices. Such a site-to-site variation is likely prevalent across hospitals not included in the study. This variation across sites also highlights the challenge with working with RWD. For example, for the Mayo Clinic sites where the diagnosis code had a high precision but low recall, may be due to testing at standalone testing facilities. Specifically, the Rochester, MN site served as high-volume drive-thru testing site for the local community. These individuals were less likely to have prior data in the Mayo EHR, and thus more likely to not have a positive test result lead to a diagnosis code in the EHR.

Fifth, important measures such as those assessing interrater reliability^27,28^, or discrimination (c-statistic) were limited by the undefined size of the true negatives, i.e., individuals without a diagnosis code who also did not have a laboratory diagnosis is challenging to define, particularly given the variation in testing thresholds and the lack of information on the universe of patients who neither underwent testing or had a diagnosis code. The precision and recall are relevant in assessing model performance, and do not depend on this information. Finally, while we pursued a limited chart review to assess the clinical presentations that underlie patterns in coding for a small number of randomly drawn cases, these may not represent all the possible reasons for the observed variation in coding. The time-intensive and subjective nature of the review by clinical experts precluded a broad validation against manual assessment.

In conclusion, the use of COVID-19 diagnosis codes misclassified SARS-CoV-2 infection status for many people, with implications for clinical research and epidemiological surveillance. Moreover, the codes had different performance across two academic health systems and identified groups with different risks of mortality. Standardization of coding practices and their validation against other data streams in the EHR is needed to allow for the use of diagnosis codes for clinical research and epidemiological surveillance of COVID-19.

## Methods

### Data sources

We used EHR-derived data from Yale New Haven Health System (Yale), a large academic health system consisting of five distinct hospital delivery networks and associated outpatient clinics located in Connecticut and Rhode Island. To evaluate the generalizability of our observations, a similar cohort was constructed in the three hospital delivery networks of the Mayo Clinic. Mayo Clinic is an academic health system headquartered in Rochester MN, with two additional destination medical centers in Phoenix, AZ, Jacksonville, FL, and several regional and critical access hospitals in Minnesota and Wisconsin.

All included study sites use an Epic EHR system. At Yale, the data from Epic were transformed into the Observational Medical Outcomes Partnership (OMOP) common data model (CDM)^13,29^. At the Mayo Clinic sites, the corresponding data fields were captured from the Epic EHR extract directly.

We used a versioned extract from March 3, 2021 and analyzed testing and discharge information from April 1, 2020, when the COVID-19 specific *International Classification of Diseases-10*^*th*^ *Edition-Clinical Modification (*ICD-10-CM*)* diagnosis was introduced, through March 1, 2021. Admissions were limited to those with a visit start date before January 31, 2021 to allow for a majority of those admitted to have been discharged^3^.

The study was approved by the Institutional Review Boards of Yale University and Mayo Clinic. Data were independently analyzed at each site.

### Cohort definitions

#### SARS-CoV-2 infection

We defined two strategies to identify SARS-CoV-2 infection from the EHR spanning all healthcare settings, the first based on diagnostic codes and the second based on laboratory testing. Our first approach relied on the identification of the specific COVID-19 ICD-10-CM diagnosis code U07.1 within the clinical record, defined based on encounter-associated diagnoses. The U07.1 code, which was introduced on April 1, 2020, was used to define SARS-CoV-2 when used either as a principal or a secondary diagnosis of COVID-19 during any healthcare encounter.

The two diagnosis-based phenotyping strategies were compared to the second approach, which was based on the presence of a positive SARS-CoV-2 PCR or antigen test within 2 weeks before or after the diagnosis encounter, to identify individuals who had documented infection. The laboratory testing did not focus on antibody testing given the testing’s limited role in the assessment of active disease.

We supplemented this assessment to include potentially related but non-specific diagnoses for severe acute respiratory syndrome (SARS) or coronavirus disease (COVID-19-related diagnoses) based on a subset of codes identified within the National COVID Cohort Collaborative (N3C) phenotype (see Supplementary Table 2)^30^.

#### COVID-19 hospitalization

We defined COVID-19 hospitalizations using two strategies. The first identified all hospitalizations with a principal diagnosis of COVID-19 (U07.1). In addition, we defined a second strategy that included individuals with a secondary diagnosis of COVID-19, but with a clinical presentation that was consistent with severe manifestations of COVID-19 defined by a principal diagnosis for acute respiratory failure, pneumonia or sepsis. This approach focused on hospitalizations that were due to COVID-19 rather than incidentally associated with a positive test for the disease during admission for an unrelated diagnosis. The principal diagnoses used in this approach are included in Supplementary Table 3. There was only a single hospitalization with a diagnosis code J12.82 that has been suggested to identify COVID-19 pneumonia^30^, and was not included in the analysis. Further, to assess validity of diagnosis codes to identify COVID-19 hospitalizations, we compared hospitalizations with COVID-19 diagnosis codes against positive SARS-CoV-2 testing 2 weeks before hospitalization through any time before hospital discharge.

### Study covariates

We defined key demographic characteristics for individuals, including age, sex, race and ethnicity. Age was defined as completed years on the day of admission, computed from their date of birth. Sex, race, and ethnicity were based on what was documented in the medical record. To evaluate the effect of coding strategies on case identification among racial and ethnic minorities, we combined racial/ethnic groups into mutually exclusive groups of Hispanic, non-Hispanic White, non-Hispanic Black, and other race/ethnicity groups^31,32^. The authors explicitly confirmed that the study fields were defined based on the same EHR data fields.

Among patients hospitalized with COVID-19, the representativeness of cohort definitions was assessed based on inhospital mortality rates across case identification strategies. Inhospital mortality was defined based on the discharge disposition of the index (first) COVID-19 hospitalization. Consistent with other studies^33–35^, we used a composite endpoint of inhospital mortality, transfer to inpatient hospice, or discharge to facility or home-based hospice to define our composite outcome.

### Statistical analyses

We compared differences in demographic characteristics using the chi-square test for categorical variables and t-test for continuous variables. We evaluated the performance of computable phenotyping of SARS-CoV-2 infection and hospitalization based on the COVID-19 diagnosis code. This was evaluated against a confirmed diagnosis of SARS-CoV-2 infection based on PCR or antigen testing. The performance of COVID-19 diagnoses to accurately identify cases of SARS-CoV-2 infection was assessed on 2 key performance measures: precision (positive predictive value) and recall (or sensitivity). Precision was defined as the proportion of patients with a COVID-19 diagnosis that had a positive PCR/antigen test for COVID-19. Recall was defined as the proportion of patients with a positive PCR/antigen test for COVID-19 who also had a diagnosis of COVID-19 among all patients who tested positive with or without a diagnosis of COVID-19. The laboratory diagnosis was chosen a surrogate of a confirmed SARS-CoV2 infection, and a confirmed COVID-19 hospitalization given the wide phenotypic variability of clinical disease and a lack of an alternative algorithmic strategy for defining clinical disease in the absence of laboratory results.

Analyses were conducted using Spark 2.3.2, Python 3.6.9, and R 3.8 (packages listed in Supplementary Table 4). All statistical tests were 2-tailed with a level of significance set at 0.05.

### Manual chart abstraction and validation

Manual chart abstraction was conducted by two clinicians independently (R.K. and W.L.S.) and focused on a sample of randomly selected charts where the diagnosis codes were discordant from laboratory results. For SARS-CoV-2 infections, ten patient charts were randomly selected from each of the following categories (total 30): (1) principal or secondary diagnosis of COVID-19, but negative laboratory diagnosis (*n* = 20), and (2) a positive laboratory diagnosis of SARS-CoV-2 without a corresponding diagnosis code (*n* = 10). Furthermore, ten additional charts were selected for patients who were hospitalized with a principal diagnosis COVID-19 and negative laboratory results, and the clinical documentation was qualitatively reviewed to evaluate the reason for the discrepancy (see Supplementary Note for details).

### Generalizability of phenotypes at Mayo Clinic

We constructed equivalent patient cohorts across the three Mayo Clinic sites in Minnesota, Arizona, and Florida using the same cohort definitions as outlined in the primary analyses. In these cohorts, we evaluated both the accuracy of the coding strategies in identifying infections with SARS-CoV-2 across care settings, and COVID-19 hospitalizations.

### Reporting summary

Further information on research design is available in the Nature Research Reporting Summary linked to this article.

## Supplementary information

Online Supplement

Reporting Summary

## Data Availability

The patient-level information is not available for public release due to restrictions in the IRB approval at each site. However, summary data required to replicate figures will be provided upon request.

## Code availability

The statistical code is available on a public repository (https://github.com/rohankhera/covid-phenotyping-share)^36^. Analyses were conducted using Spark 2.3.2, Python 3.6.9, and R 3.8 and a complete list of analytic packages listed in Supplementary Table 4.
