# Supplementary material for: A multicenter evaluation of computable phenotyping approaches for SARS-CoV-2 infection and COVID-19 hospitalizations
Source: NPJ Digit Med. 2022 Mar 8;5:27. doi: 10.1038/s41746-022-00570-4 (PMC8904579; doi:10.1038/s41746-022-00570-4)

**Khera et al. “A Multicenter Evaluation of Computable Phenotyping Approaches for SARS-CoV-2 Infection and COVID-19 Hospitalizations”**

**ONLINE SUPPLEMENT**

## SUPPLEMENTARY NOTE: CHART ABSTRACTION

Two clinicians, one from internal medicine (RK), and another from laboratory medicine (WS), both with experience with Epic, independently reviewed all selected charts using a designed format. They reviewed all encounters within 2 weeks before or after the reported of laboratory test or a diagnosis code.

The review process was standardized and followed the following format. We reviewed all notes (clinic, hospital admission, hospital progress, and discharge summary, along with nursing and telephone notes) for results of laboratory testing and diagnosis codes associated with each encounter. In addition, laboratory testing records were reviewed for both those performed in the health system a. The notes and scanned documentation were reviewed for positive SARS-CoV-2 testing at outside hospitals. Based on the review of the data, a determination was made whether a SARS-CoV-2 antigen or polymerase chain reaction (PCR) test was performed and whether the test indicated an infection. The nature of the encounter that prompted the documentation of a diagnosis code for COVID-19 was also noted.

A true positive laboratory test and clinical diagnosis were considered when the diagnosis code and SARS-CoV2 test were both positive. If a diagnosis was not recorded but a positive test was noted, they were considered false negatives. In both these instances, the completion of laboratory tests at other institutions or a strong clinical suspicion of COVID-19 in the presence of a negative SARS-CoV-2 test were considered positive laboratory tests, i.e. the patients were considered to have had an infection. Therefore, the absence of a diagnosis in such a patient would be considered a false negative. In contrast, among patients with only a diagnosis code without a positive test across encounters, outside hospitals, or without strong clinical suspicion, documented in the chart were considered false positives. In our review, we recognized instances of this when a COVID-19 diagnosis code was included in the chart to support the testing for SARS-CoV-2 for a telephone or clinic encounter when patients requested screening without symptoms, but the test was noted to be negative without any mention of clinical disease.

**Supplementary Table 1:** Monthly counts of non-specific coronavirus associated diagnoses and those with a laboratory diagnosis in the Yale New Haven Health System (i.e. positive laboratory test for SARS-CoV-2 nucleic acid or antigen).

| | 2020 | | | | | | | | | 2021 | |
|---|---|---|---|---|---|---|---|---|---|---|---|
| | Apr | May | Jun | Jul | Aug | Sep | Oct | Nov | Dec | Jan | Feb |
| B97.21 | 29 | 16 | 3 | 17 | 4 | 4 | 14 | 15 | 16 | 12 | 3 |
| B97.29 | 146 | 172 | 159 | 206 | 139 | 87 | 49 | 90 | 64 | 25 | 6 |
| B34.2 | 205 | 67 | 40 | 86 | 64 | 68 | 96 | 338 | 200 | 52 | 15 |
| J12.81 | 37 | 33 | 11 | 3 | 2 | 3 | 5 | 12 | 20 | 25 | 11 |
| B97.27 + Lab diagnosis | 9 | 9 | 1 | 3 | 1 | 1 | 4 | 2 | 5 | 4 | 1 |
| B97.29 + Lab diagnosis | 43 | 29 | 10 | 12 | 21 | 16 | 11 | 51 | 22 | 8 | 2 |
| B34.2 + Lab diagnosis | 75 | 21 | 9 | 5 | 6 | 3 | 4 | 61 | 63 | 19 | 3 |
| J12.81 + Lab diagnosis | 20 | 15 | 6 | 1 | 1 | 3 | 2 | 7 | 11 | 16 | 8 |

**Supplementary Table 2: Non-specific coronavirus diagnosis codes.**

| ICD-10-CM Code | Description |
|---|---|
| B97.21 | SARS-associated coronavirus as the cause of diseases classified elsewhere |
| B97.29 | Other coronavirus as the cause of diseases classified elsewhere |
| B34.2 | Coronavirus infection, unspecified |
| J12.81 | Pneumonia due to SARS-associated coronavirus |

*Codes included in the initial cohort selection strategy by the National COVID Cohort Collaborative (N3C). Abbreviations: ICD-10-CM: International Classification of Diseases, Tenth Revision, Clinical Modification; SARS – severe acute respiratory syndrome.

**Supplementary Table 3:** Primary diagnosis codes for severe COVID-19 manifestations or non-specific coronavirus infection recorded in our study population with a secondary diagnosis of COVID-19 (U07.1).

| ICD-10-CM Code | Description |
|---|---|
| A41.89 | Other specified sepsis |
| A41.9 | Sepsis, unspecified organism |
| B34.2 | Coronavirus infection, unspecified |
| J12.89 | Other viral pneumonia |
| J18.9 | Pneumonia, unspecified organism |
| J80 | Acute respiratory distress syndrome |
| J96.01 | Acute respiratory failure with hypoxia |
| J96.21 | Acute and chronic respiratory failure with hypoxia |

Abbreviation: ICD-10-CM: International Classification of Diseases, Tenth Revision, Clinical Modification

**Supplementary Table 4:** Key analytic packages and software used in the analysis.

| Library | Version | Ref/Link |
|---|---|---|
| Spark | 2.3.2 | https://spark.apache.org/docs/2.3.2/ |
| Python | 3.6.9 | https://www.python.org/downloads/release/python-369/ |
| Python: Scikit Learn | 1.0.1 | https://scikit-learn.org/stable/ |
| Python: Numpy | 1.17.0 | https://docs.scipy.org/doc/ |
| Python: Scipy | 1.7.1 | https://docs.scipy.org/doc/ |
| Python: Pandas | 1.3.4 | https://pandas.pydata.org/docs/ |
| Python: vcg | 1.3.0 | https://pypi.org/project/vcg/ |
| R | 3.8 | https://www.r-project.org |
| R library: VCD | 1.4.9 | https://cran.r-project.org/web/packages/vcd/index.html |
| R library: hmisc | 4.6-0 | https://cran.r-project.org/web/packages/Hmisc/index.html |

**Supplementary Figure 1:** Diagnostic groups for SARS-CoV2 infection across the study period, (A) April 2020 to August 2020, (B) September 2020 to March 2021

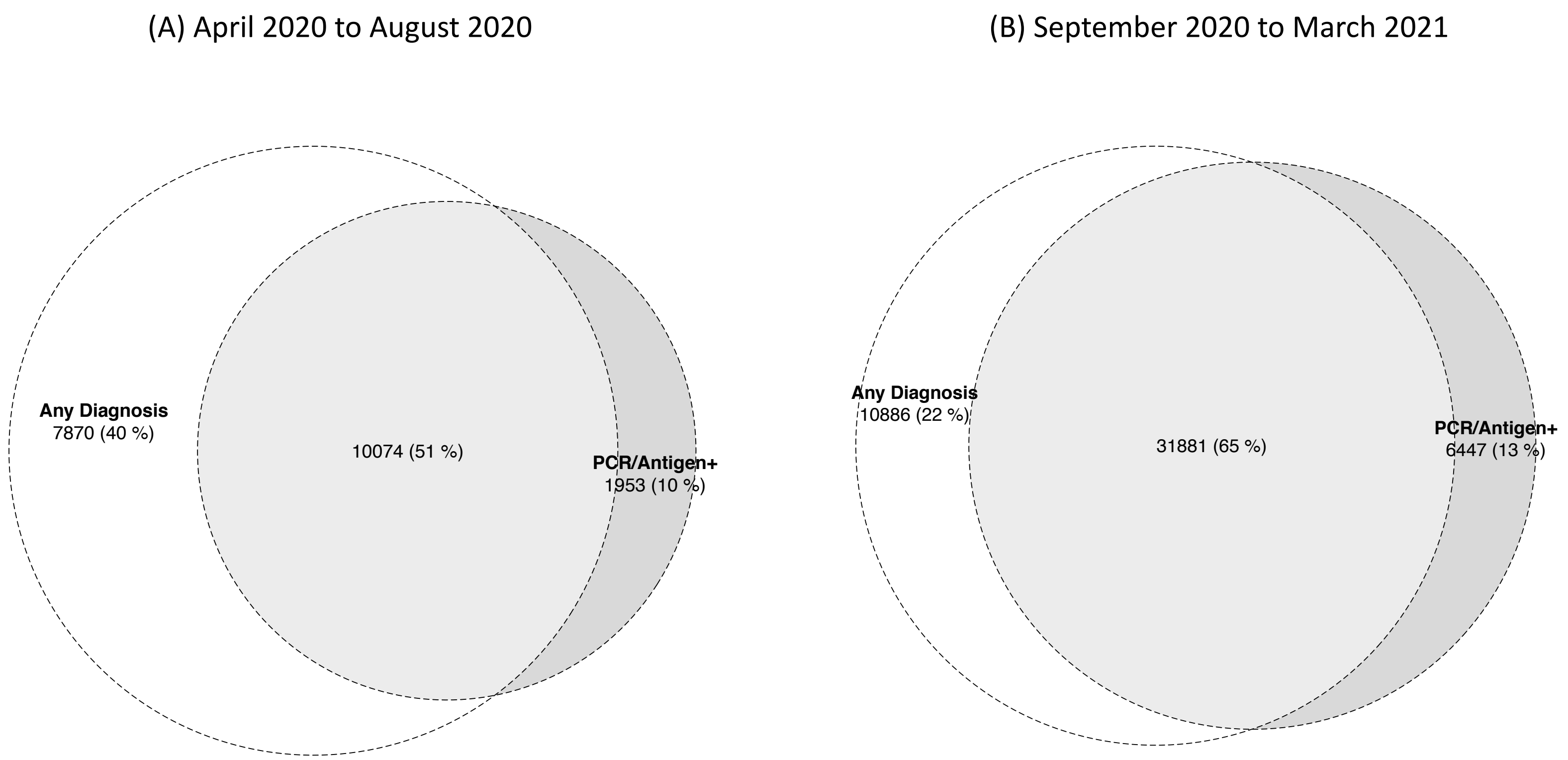

Supplement: Supplementary file 1 — Online Supplement [file 41746_2022_570_MOESM1_ESM.pdf]
